# Estimating the value of tropical coastal wetland habitats to fisheries: Caveats and assumptions

**DOI:** 10.1371/journal.pone.0215350

**Published:** 2019-04-17

**Authors:** Kátya G. Abrantes, Marcus Sheaves, Jakob Fries

**Affiliations:** 1 College of Science and Engineering, James Cook University, Townsville, Queensland, Australia; 2 TropWATER (Centre for Tropical Water and Aquatic Ecosystem Research), James Cook University, Townsville, Queensland, Australia; Tanzania Fisheries Research Institute, UNITED REPUBLIC OF TANZANIA

## Abstract

Habitat valuation can provide an objective basis for the prioritisation of conservation and restoration actions. The attribution of fisheries production to particular habitat units is an important measure of value, but is difficult to estimate. Using the case study of habitat use by juvenile banana prawns in a tropical estuary, we assessed the potential to produce valid value estimates at two spatio-conceptual scales: estuary reach and whole estuary. Additionally, we also explore the potential to produce meaningful value estimates at the scale of whole estuary contribution to the offshore fisheries stock. A diversity of potential and actual sources of error and logical problems means that quantification at any scale is at best of uncertain validity and produces estimates that are likely to produce unreliable results if treated as quantitative inputs to production models. Estimates for the whole estuary were the most viable, although still requiring substantial assumptions that may or may not be reasonable in particular situations. Estimates for individual habitats required the unreasonable assumption of limited prawn movement, while estimates of contribution of an estuary to the fishery required difficult-to-obtain and usually unavailable information. Because low occupancy habitats can have trophic value, we also used stable isotope analysis to assess the importance of mangroves and saltmarshes to prawn nutrition. No particular habitat was of critical trophic importance, again suggesting that the habitat-production link is most usefully assessed at the whole-of-estuary scale. While valuable and required to support targeted ecosystem management and protection and restoration efforts, valid estimates of the contribution of particular units to fisheries are likely to be unachievable in many situations.

## Introduction

### Measuring the value of wetland habitats

Estuaries and coastal wetlands have always been important to human societies, fulfilling a variety of ecological services that are critical to human livelihoods and food security such as fisheries production, providing nursery grounds for important species, and filtering and detoxification [1]. Although healthy coastal wetlands have significant intrinsic ecological values that underpin basic human needs, modification of wetlands brings other important socio-economic advantages (e.g. urbanisation, port construction, construction of tidal exclusion bunds to increase area for agriculture, etc.). Since ecosystem management decisions are often based on economic evaluation, being able to attribute economic value to these systems and their habitats in a quantifiable way is an increasingly important goal [2, 3]. Accurate and valid measures of habitat value are critical for many reasons. For instance, valid estimates are needed to ensure the significance of estuaries and coastal wetlands are given appropriate weight in policy and management decisions [2], and ensuring that offsets and ecosystem repair can be prioritised and their outcomes measured [4, 5].

### The banana prawn

The banana prawn (*Fenneropenaeus merguiensis*, Penaeidae) is one of the most important fishery species in tropical northern Australia, where its biology and ecology have been researched extensively. Juvenile *F*. *merguiensis* are strongly associated with mangrove estuaries [6, 7], and *F*. *merguiensis* catches are correlated with the extent of mangrove forests along adjacent coasts ([8], but see Lee [9] for an alternative suggestion), suggesting this species provides a useful model for the study of habitat-production relationships. Such a model is likely to have broad applicability because penaeid prawns are commercially important throughout the world due to their high densities, very high reproductive output, and fast growth rates that allow harvesting within a year [10–12]. Moreover, the use of coastal nurseries by *F*. *merguiensis* is typical of many penaeids [13], with adults occurring in near- and offshore waters, and larvae that migrate to utilise estuarine, seagrass, marine wetland or other coastal nursery areas [7, 14].

Production (defined as the total expected increase in biomass over time for a population [15]) is a critical parameter for estimating the economic value attributable to a particular unit of habitat. However, despite the potential usefulness of *F*. *merguiensis* in developing a quantified understanding of the fisheries value of coastal wetland nurseries, developing a valid production model is complex. A diversity of factors synergistically affect prawn catches (e.g. [16, 17]). Because many influences other than the presences of mangroves drive abundances [18], and that fisheries catches typically occur in offshore habitats that are distant from the coastal juvenile habitats, it is intrinsically difficult to estimate the value for fisheries production that arises from the preservation or restoration of a particular unit of e.g. mangrove, coastal salt marsh or mudflat habitat, and therefore difficult to assess the monetary value per area of the particular habitat units.

Even within a region, the complex of biological, physio-chemical and climatic factors that determine recruitment, growth and survival [16, 19] cause landings to vary substantially among- and within-years and among locations [20]. Typically, the abundance of commercial-sized penaeid prawns is predicted using one of three approaches: 1) adult abundance is related to biological factors such as the abundance of post-larvae or juveniles in related habitats (e.g. [21, 22]), 2) adult abundance is related to environmental parameters (e.g. rainfall, habitat type and availability, temperature) (e.g. [23, 24]), or 3) stock-recruitment models are used to relate the abundance of penaeids at the reproductive stage in one generation to the numbers entering the fishery in the following generation (e.g. [12, 25]). Where comprehensive data are available, models that combine the three approaches have been developed [16]. Thus, it is clear that the abundance of early life-history stages and the ecological and physio-chemical conditions prevailing in nursery habitats are critical factors affecting adult abundance and, therefore, potential fisheries output. In face of global increase in coastal habitat degradation due to various anthropogenic actions (e.g. agriculture, urbanisation), it is imperative to identify and attribute a value to the most important habitats supporting the early life-history stages of penaeid prawns, so that these can be prioritised in conservation and/or restoration projects, ensuring continuing profitable fisheries [4].

#### Production estimates

The first step in addressing habitat valuation in terms of *F*. *merguiensis* fisheries output would be to determine the importance of the different juvenile habitats. Measures of production are particularly useful in habitat valuation [26] because they attribute a value to a habitat unit by detailing the amount of biomass produced from that unit over a specific time period. Although basic information on *biomass density* (i.e. biomass per unit area) is available for *F*. *merguiensis* for several estuaries in North Queensland, Australia (e.g. [18]; Sheaves, Abrantes, unpubl. data), substantial additional data are needed before biomass density can be converted to valid estimates of total biomass and/or production, particularly if the purpose is to link production to specific habitat units. Indeed, biomass density needs to be estimated for the different habitats at scales that specifically reflect the reality of the distribution of particular habitat types. These results then need to be combined with detailed habitat mapping (including topography, vegetation and bathymetry). In addition, growth rates, size-related mortality rates and recruitment and emigration rates need to be established and integrated into modelling to produce viable estimates of production [27, 28]. Beyond that, studies are needed to develop the best possible estimates of biomass density for habitats that cannot be sampled using conventional gears.

Although production estimates provide the most complete picture of the fisheries value of a habitat or area, in many cases the necessary base data on parameters such as growth, mortality and emigration rates are unavailable and unlikely to be obtained/developed, because they are time-consuming and logistically difficult to collect, and because the dynamics of species populations relative to individual target habitats prevents the development of valid estimates. Consequently, it is often more reasonable and profitable to direct work towards ensuring that estimates of *biomass density* for the different habitat units are of the highest quality possible. Although not integrated over time, and so not providing direct information on the increase in biomass per time, biomass density estimates are more easily achievable and, as long as their limitations as snapshots in time are recognised, can provide useful relative measures of habitat value that are easily understood and communicated. For a particular point in time, biomass density estimates have the potential to be used as a valid basis for evaluating the contributions from estuary and coastal wetland habitats, and can provide the basis understanding of how those contributions are likely to change under different scenarios.

### Sources of nutrition

A second approach to understanding the value of coastal wetlands is to establish their importance in supporting nursery ground food webs. Again, *F*. *merguiensis* are a useful focal species for food web studies. They are key components of food webs of coastal wetlands and estuaries, where they occupy low trophic levels, feeding mostly on detritus (up to 75%) of various origins and small benthic invertebrates such as crustaceans and gastropods [29, 30]. Stable isotope-based studies have been used to identify the ultimate sources of nutrition to *F*. *merguiensis* juveniles in several North Queensland systems including rivers [31], estuarine creeks [32], floodplain pools [33–35] and semi-enclosed coastal channels [36]. Although those studies suggest that mangroves can be important contributors to juvenile nutrition, no study has confirmed if this source is of critical importance or if *F*. *merguiensis* juveniles can exclusively rely on other sources when mangroves are not present. Consequently, a critical link in estimating habitat value is missing.

Stable isotope analysis of carbon (δ^13^C) and nitrogen (δ^15^N) are typically used in coastal food web studies because (1) they often differ among different types of primary producers (e.g. seagrass, mangroves, microalgae; particularly for δ^13^C) [37, 38] and (2) because they change predictability as they are passed on from food source to consumer [39–41]. Stable isotope analysis can therefore be used to determine if mangroves and/or saltmarsh are crucial sources of nutrition to *F*. *merguiensis* juveniles in North Queensland estuaries, so that the trophic importance of these habitats can be evaluated.

In the present study, we use the banana prawn, *F*. *merguiensis*, from one estuary as a case study to evaluate the potential for producing valid estimates of the value of estuarine habitats to nekton in relatively data-poor situations. First, we use measurements of numerical density and biomass density to assess the suitability of the currently available methods of estimating total abundance, biomass and production to estimate the value of coastal wetland and estuarine habitats to nekton. In particular, we evaluate the potential of producing valid and valuable estimates, in terms of logical constraints and estimated error structure, at three spatio-conceptual scales: (i) estuary reach, (ii) the whole estuary, and (iii) the contribution of the estuary to the offshore exploited stock. Note that even if/when high quality estimates of biomass production are available, more information is likely to be required to truly assess value. This is because even habitats with low occupancy can have a trophic importance through the provision of essential nutrients needed for growth and development. Consequently, we also use δ^13^C and δ^15^N to investigate the trophic importance of mangroves and saltmarshes for *F*. *merguiensis* juveniles in four North Queensland estuaries.

## Methods

### Evaluating F. merguiensis abundance and biomass

The productivity component of this study used *F*. *merguiensis* from Alligator Creek as a case-study. Alligator Creek is a short and narrow (maximum width ~150 m) estuary typical of Australia’s wet-dry tropics. Details of this site can be seen in Sheaves [42]. The wet-dry tropical climate is characterised by distinct and short wet seasons and long dry seasons when very little rainfall occurs. Alligator Creek is bordered by a mangrove fringe for most of its length, with extensive mangrove forests developed in areas of lower elevation, and saltmarsh and saltpan occupying higher intertidal areas landward of mangroves. Tides are semi-diurnal (maximum range ~4 m). Sampling was conducted in three estuarine reaches: downstream, mid-estuary and upstream, each sampled over a length of ~1.5 km. The downstream reach was the area immediately inside the estuary mouth, the upstream reach was the limit of navigation of the 4.3 m sampling vessel, and the mid-estuary was approximately half-way between the downstream and upstream reaches. Sampling was conducted at low tides (channel depth <~1.5 m), in both spring and neap tide phases. The lower part of the tide was used as animals are then forced out of the structured edge habitats (i.e. mangrove/saltmash habitats, which are more difficult to sample) and into the main channel where they concentrate along banks and habitat edges [43]. Water depths sampled ranged between ~0.1 and ~1 m in both the upstream and mid-estuary reaches, and ~0.1 and ~2 m in the downstream reach.

Sampling was conducted over the 2015–2016 wet season (December 2015 to April 2016), to encompass the period of high *F*. *merguiensis* juvenile abundance in northern Australia [44, 45]. Prawns were captured during 11 trips with a 5 mm mesh monofilament draw-string cast net (sampling diameter: ~2.4 m; sampling area: ~4.5 m^2^) deployed from the small sampling vessel. To maximise consistency, sampling was carried out by a single experienced operator. Sampling was conducted along muddy estuary banks clear of structural complexity, where nets could be deployed without snagging, during the lower part of the tides when animals are forced out of the edge habitats and into the main channel. During each trip, 15, 20 or 30 cast net replicates were taken along the creek banks (≤~2m from creek margin) at each of the three reaches. Because *F*. *merguiensis* juveniles occur mostly along the shallow, 2 m wide edges [43], we focus our results and assessments on densities (per net) within the 2 m creek margins, rather than mean densities over the overall creek area. All prawns collected were stored on ice. In the laboratory, prawns were identified and *F*. *merguiensis* counted, measured and weighed. Numerical density (in ind.net^-1^) and biomass density (in g.net^-1^) were then calculated for each reach, and used to estimate total abundance and total biomass per reach. Here, measured densities per net were used as representative of the area along the 2 m wide creek margins (downstream: 19,027 m^2^; midstream: 26,920 m^2^; upstream: 11,289 m^2^), as *F*. *merguiensis* typically occur along shallow water creek edges [43]. The remaining estuarine area (i.e. the middle of the creek, beyond the 2 m wide edges) was considered to contain few prawns, i.e. densities were considered to be close to zero (R. Johnston pers. com.). The relative abundance-by-length was also analysed for each reach and trip to identify movements among reaches and recruitment/emigration events.

### Statistical analysis of catch data

**Fig 1:**
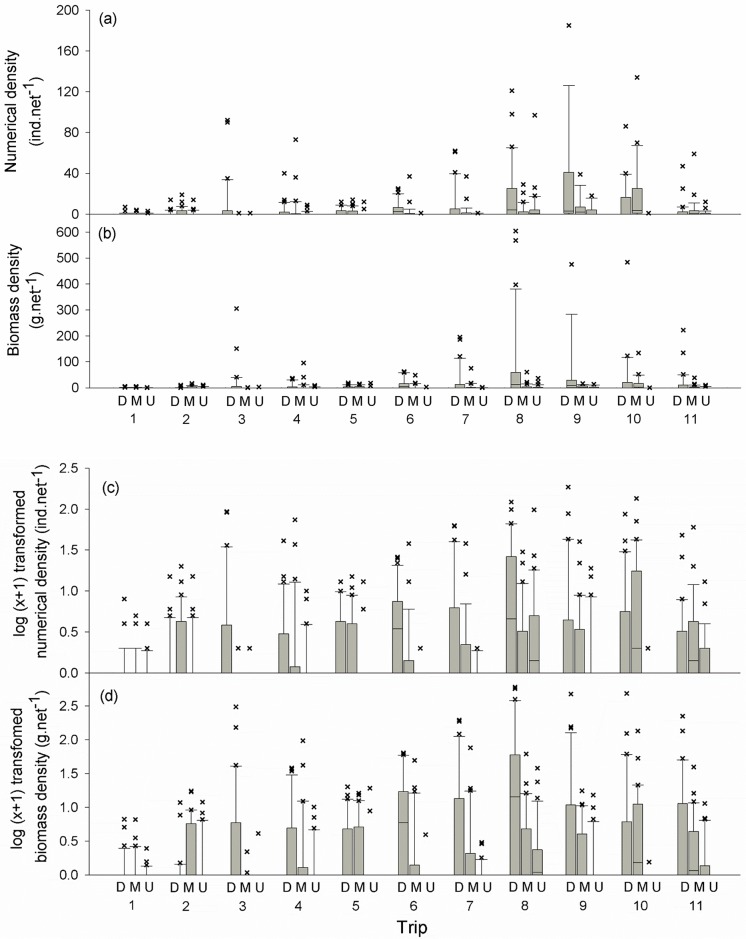
*Fenneropenaeus merguiensis* numerical density and biomass density. Box and whisker plots show the median (line within the boxes), interquartile range (boxes), 10^th^ and 90^th^ percentiles (whiskers) and outliers (x) of (a) numerical density (number of individuals per net) untransformed, (b) and biomass density (weight per net) untransformed, (c) numerical density (number of individuals per net) log (x+1) transformed, and (d) biomass density (weight per net) log (x+1) transformed, of *F*. *merguiensis* collected in Alligator Creek over the 2015–2016 wet season (trips 1 to 11; see trip dates in Fig 3). Estuary reaches: D = downstream; M = mid-estuary; U = upstream reach.

**Fig 2:**
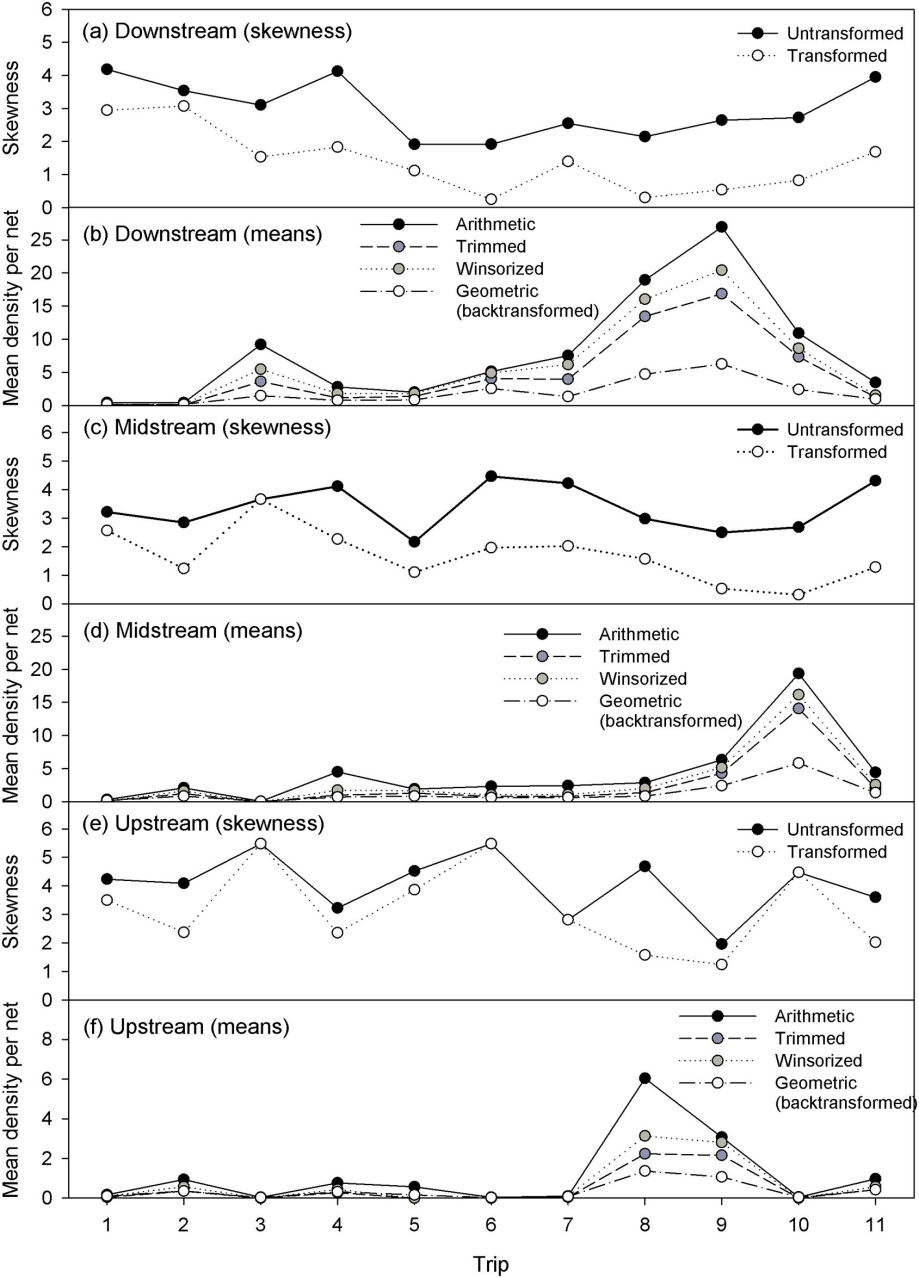
Mean *Fenneropenaeus merguiensis* densites and skewness of density data. Mean *Fenneropenaeus merguiensis* densities (per net) and skewness of density data for the downstream (a, b), midstream (c, d) and upstream (e, f) reaches of Alligator Creek estuary over the 2015–2016 wet season (trips 1 to 11; see trip dates in Fig 3). Mean densities (b, d, f) were calculated as raw arithmetic means, trimmed means (based on excluding a predetermined 5% of points from both ends of the data), Winsorized means (with Winsorizing applied to a predetermined 5% from both ends of the data), and log (x+1) transformed means. Skewness (a, c, e) was calculated using untransformed and log (x+1) transformed data.

Catch data were extremely variable, with a preponderance of zero catches (Fig 1a), medians of numerical (Fig 1a) and biomass (Fig 1b) density at or close to zero and many outliers apparent for all trips and reaches (Fig 1a and 1b), resulting in extremely positively skewed distributions (skewness >1; Fig 2a, 2c and 2e). Because of the preponderance of zeros (Fig 1a), the skewness situation was not greatly improved by transformation (Figs 1c, 1d, 2a, 2c and 2e). Consequently, estimates of the mean from these data are very dependent on the approach used. We calculated means in four ways: raw arithmetic means, trimmed means (based on excluding a predetermined 5% of points from both ends of the data to reduce the effect of outliers), Winsorized means (with Winsorizing applied to a predetermined 5% from both ends of the data) [46, 47], and log (x+1) transformed means (Fig 2b, 2d and 2f). Each of these methods produced quite different estimates of the mean, with the arithmetic mean consistently leading to the highest estimate (Fig 2b, 2d and 2f). Because of the uncertainty of the values of all mean estimates (Figs 1 and 2), we report and focus on arithmetic means, noting that these have no validity beyond representing the maximum mean estimates and are therefore unreliable as inputs to quantitative analyses or modelling.

### Sources of nutrition

*Fenneropenaeus merguiensis* juveniles and a range of available primary producers were collected from four representative systems in North Queensland: Deluge Inlet, Cocoa Creek, Doughboy Creek, and two semi-isolated floodplain pools in the Ross River estuary, one surrounded by mangrove vegetation and one by saltmarsh, with only a few small mangrove trees present (see [32, 48, 49] for details on study sites). Deluge Inlet is a mangrove-dominated system that flows into the Hinchinbrook Channel [49]. Seagrass beds occur at the mouth of the Inlet and in the Channel. Cocoa and Doughboy creeks are short and narrow mangrove-fringed systems typical of the North Queensland coast. Mangrove forests are more extensive at Doughboy Creek, with a percentage cover of 50% compared to 19% at Cocoa Creek (percentage cover calculated for the area within 1 km from the creek margins) [32]. Saltmarsh occurs landward of the mangrove fringe and seagrass is also present at the mouth of Cocoa Creek. The two Ross River floodplain pools are relatively small and shallow (<1 m at low tide) and are intermittently connected at spring tides to other pools and to the main estuary through narrow channels [48]. These four different systems were chosen to represent the range of system types and habitats available to *F*. *merguiensis* juveniles in North Queensland.

Juveniles analysed ranged in size between 30 and 45 mm TL, with exception of the Hinchinbrook Channel, where it was only possible to collect larger (50–55 mm TL) juveniles. Details of animal collections and stable isotope sample processing and analysis can be found in Abrantes and Sheaves [33, 36] and Abrantes et al. [32].

To quantify the importance of mangroves, saltmarsh and seagrass (i.e. of the wetland habitats generally considered in management) to *F*. *merguiensis* juveniles, stable isotope-based Bayesian mixing models were run, using the package simmr (Stable Isotope Mixing Model in R v.3 [50]) in R [51]. Details of this model can be found in Parnell et al. [50, 52]. Trophic discrimination factors of 1.0 ± 0.5‰ (± SD) for δ^13^C and 2.8 ± 0.5‰ for δ^15^N were used, as appropriate for non-acid treated muscle tissue [41], and juveniles were considered to be of trophic level of 2.5 [33]. Results relating to the importance of mangrove and saltmarsh were considered as indicators of the likely trophic value of these habitats to *F*. *merguiensis* juveniles.

All research procedures received the approval from the Animal Ethics Committee, James Cook University (Ethics Approval A852_03 and A2184).

## Results

### Evaluating F. merguiensis abundance and biomass

High numbers of replicates with zero values (i.e. with no individuals captured; S1 Table) rendered even robust estimates of central tendency of doubtful value (see above). We report arithmetic means as estimates of the maximum values of parameters but again emphasise that, because of the poor quality of the estimates, these values are inappropriate as inputs for further quantitative analyses or modelling. Rather, they should be considered the best available indices of numerical and biomass densities.

**Fig 3:**
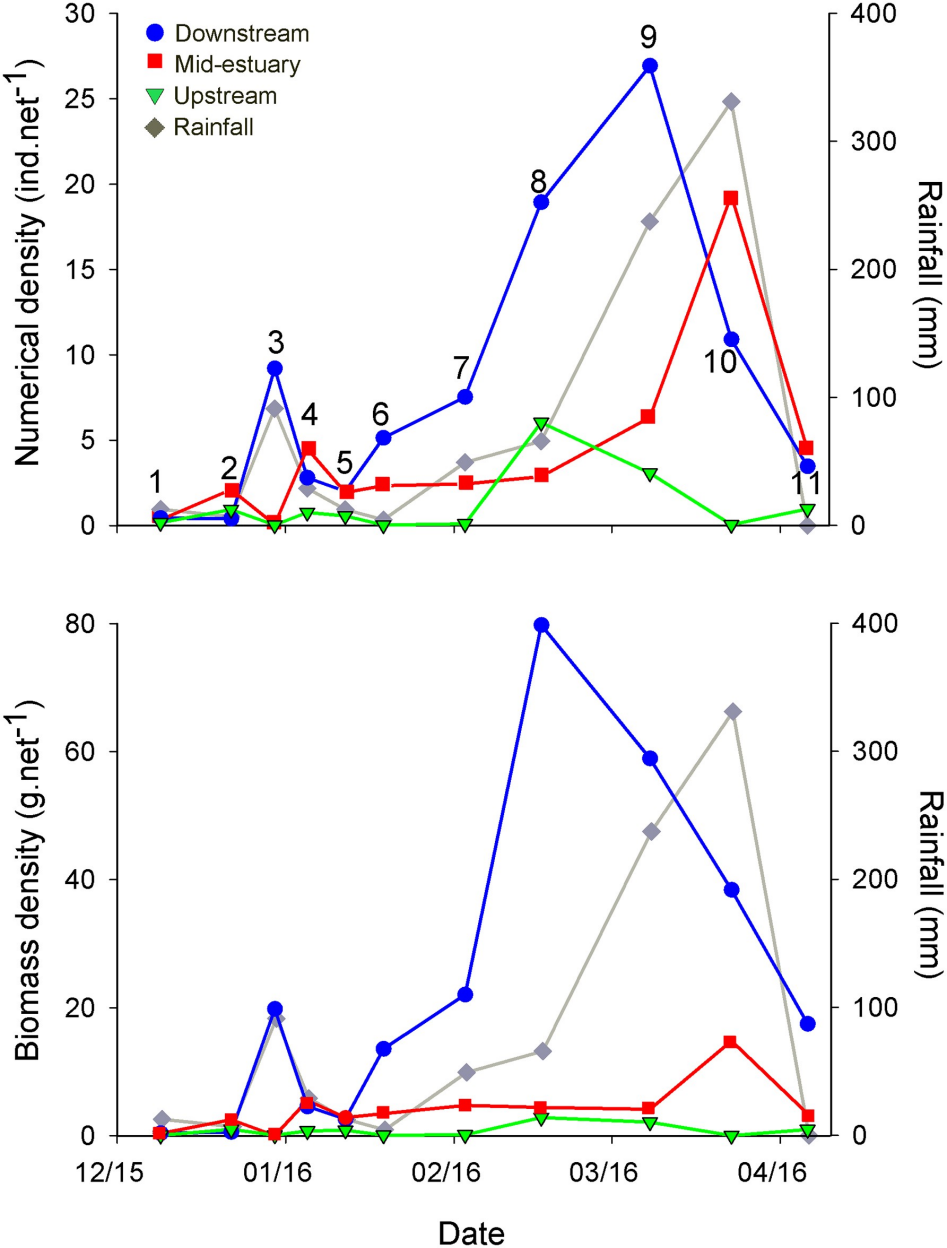
*F*. *merguiensis* density and biomass. Mean (arithmetic mean) numerical density (top) and biomass density (bottom) of *F*. *merguiensis* in Alligator Creek over the 2015–2016 wet season, and rainfall recorded for Townsville (the closest weather station, ~27 km away) for the same period (Bureau of Meteorology, www.bom.au, accessed 09/02/17). For rainfall, values correspond to the total rain (mm) that fell between the respective date and the previous sampling trip, apart from trip 1, for which rainfall of the previous 15 days is indicated. Trip numbers are indicated in the top panel.

Both mean numerical density and biomass density varied greatly among trips and reaches (Figs 1 and 3; S1 Table). In general, few prawns were captured in the first two trips (mid December 2015), but catches increased from the third trip onwards (Figs 1 and 3). Catches were typically much higher in the downstream reach than in the mid-estuary and the upstream reaches (Fig 3).

As mentioned above, despite the large number of replicates taken at each reach/time (up to 30), there was very high variability in numerical and biomass density among replicate samples (Figs 1 and 2; S1 Table), indicating substantial heterogeneity in spatial distribution. For example, in trip 9 (08/03/16), the trip with highest downstream numerical density (see Figs 1a, 2b and 3), downstream catches ranged from 0 to 185 ind.net^-1^ (Fig 1a) even though the different replicate samples were collected from the same type of habitat. If the 90^th^ percentile of numeric density (126 ind.net^-1^; Fig 1a) was considered as representative of the maximum that could be supported by the overall downstream edge area (19,027 m^2^) at that time, it would lead to an estimated total abundance of 533,607 individuals, a value much higher than that calculated using the (arithmetic) mean density of 27 ind.net^-1^ (~114,000 individuals). Similarly, if the 90^th^ percentile of biomass density (283.4 g.net^-1^; Fig 1b) was considered as representative of what that area could provide at best at that time, it would result in an estimated total biomass of ~1,196 kg, again in high contrast to the biomass calculated using the arithmetic mean (249 kg). Note also that the more robust estimates of central tendency (trimmed, Winsorized and log (x+1) transformed means) consistently led to values lower than the raw arithmetic means (Fig 2b, 2d and 2f). So, while measures such as the 90^th^ or 95^th^ percentile could be considered to be useful to estimate what a habitat could provide at best, these measures would only be valuable if good quality density estimates, with little variability, were available.

In the downstream reach, mean numerical density increased towards the peak of the rainy season (March 2016; trip 9), up to a maximum of 27 ind.net^-1^ (Fig 3). As mentioned above, this leads to an estimated abundance of ~114,000 individuals for the overall downstream area. This mean numerical density of 27 ind.net^-1^ corresponds to 6 ind.m^-2^ for the 2 m band along the creek edges, but only to ~0.3 ind.m^-2^ in relation to the overall downstream reach area (including the area >2 m away from the margins), as very few *F*. *merguiensis* occur beyond the 2 m-wide margins [43].

As with numerical density, downstream biomass density also increased from very low mean values (<5 g.net^-1^) at the end of 2015 to a maximum of ~80 g.net^-1^ (corresponding to ~337 kg for the overall downstream reach) in February 2016 (trip 8; Fig 3), likely because of an increase in prawn abundance combined with growth of individuals. This mean biomass density of 80 g.net^-1^ corresponds to 17.8 g.m^-2^ along the margins, but to only 0.8 g.m^-2^ if considering the overall downstream reach area, again illustrating the importance of the use of adequate methodological approaches and adequate results presentation and interpretation. For example, if the cross-creek distribution of *F*. *merguiensis* juveniles [43] was not known, replicate samples would likely be taken from different distances from the edge, including in mid-creek, and mean values would be calculated based on samples from all areas, leading to erroneous density, abundance and biomass estimates. This also shows the importance of factors such as creek width and edge sinuosity for adequate numerical/biomass density estimates.

**Fig 4:**
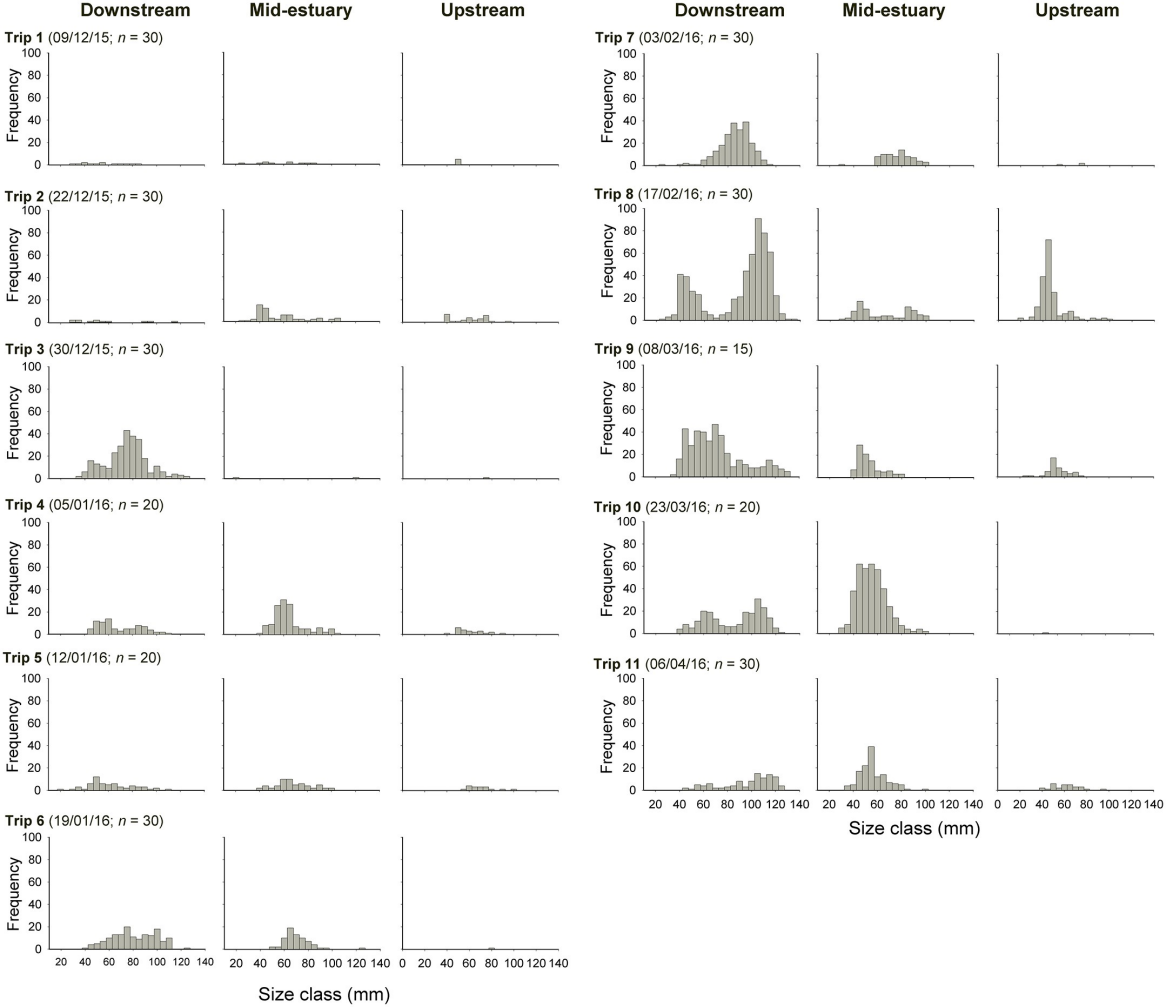
*F*. *merguiensis* size frequency distribution. Size frequency distribution of *F*. *merguiensis* captured in the downstream, mid-estuary and upstream reaches of Alligator Creek between December 2015 and April 2016 (i.e. over the wet season). Sample size, in brackets, is also indicated for each trip, and corresponds to the number of replicates taken from each of the three reaches.

After the peaks in downstream numerical and biomass densities, values decreased sharply (Fig 3), probably as a result of emigration of larger juveniles out of the estuary, a likely response to heavy rainfall, a known driver of *F*. *merguiensis* emigration from estuaries [44, 45] (Fig 3). Although this emigration resulted in a decrease in biomass from trip 8 to trip 9, numerical density increased beyond the date of peak biomass density, probably due to continuing recruitment of young individuals into the estuary, explaining why the peak in biomass density occurred before the peak in numerical density (Figs 1 and 3). Indeed, two distinguishable cohorts were present in trip 8 (17/02/16) (Fig 4; see size structure data in S2 Table), but the largest (>~100 mm total length (TL)) cohort was almost completely absent on the following trip (trip 9, 08/03/16), implying that many larger individuals moved out of the estuary with the rains that fell in the first part of March (see Fig 3), leading to a decrease in biomass density (Fig 3).

There was also a smaller peak of rainfall at the end of December 2015 (trip 3; Fig 3), that again coincided with a small peak in *F*. *merguiensis* numerical (mean 9 ind.net^-1^) and biomass density (mean 20 g.net^-1^) for the downstream reach (Fig 3). This also coincided with density dips in the mid-estuary and upstream reaches, suggesting that animals moved from the upstream areas to the downstream reach, before some moving out of the estuary and others dispersing again through the more upstream areas. This movement was likely due to the reduction in salinity in the more upstream parts of the estuary due to rainfall that occurred between trips 2 and 3 (see Fig 3). Note that salinity changes due to rainfall are less pronounced at the downstream areas due to effective tidal mixing. In trip 4, it can also be seen that the largest sizes (>~70 mm TL) that were present in trip 3 are no longer present in abundance in any of the three reaches in trip 4 (Fig 4), suggesting emigration of those larger individuals out of the estuary after the rains.

For the mid-estuary, although both numerical density and biomass density were typically much lower than in the downstream reach, a similar pattern was present, with both parameters increasing towards the peak of the wet season, and decreasing after the main rains (Fig 3). In the upstream reach, mean numerical and biomass density were very low, generally <5 ind.net^-1^ and <3 g.net^-1^ respectively (Fig 3), leading to an estimated total abundance of <3,000 juveniles, weighing ≤4 kg, for the overall upstream reach area (11,289 m^2^). Only in trips 8 (17/02/16) and 9 (08/03/16) were the estimated total upstream abundance (15,136 and 7,694 and individuals, respectively) and biomass (7.2 and 5.3 kg respectively) high, suggesting a peak in abundance and biomass at this time (February-March), possibly prior to emigration from the estuary.

For the overall Alligator Creek estuary area, based on the total abundance/biomass estimates and the total area of the 2 m wide margins for each reach, we estimate a maximum abundance of ~162,000 individuals (trip 10) and a maximum biomass of ~371 kg (trip 8).

### Sources of nutrition

**Fig 5:**
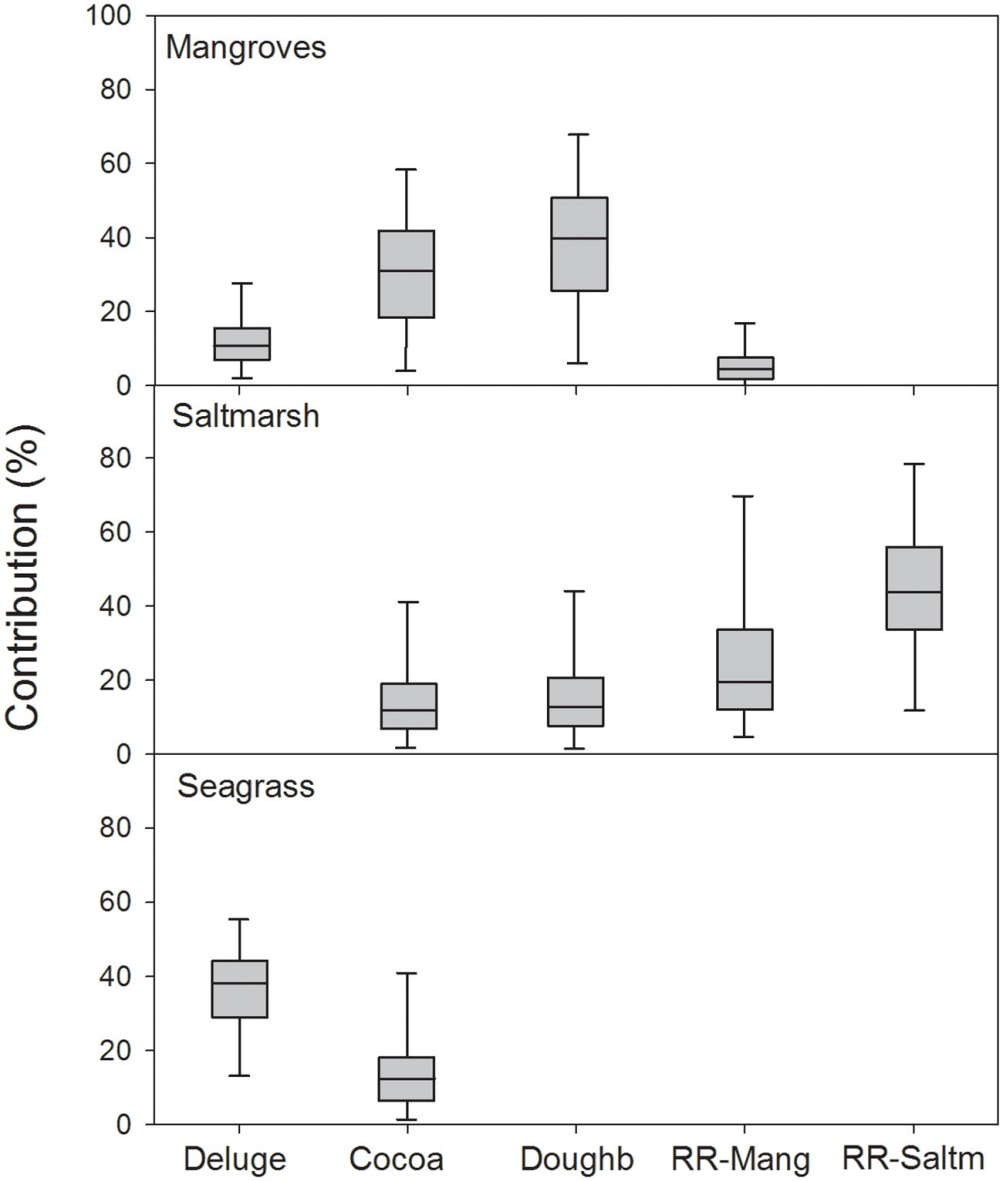
Mixing model results. Bayesian mixing model results showing the median (line within boxes), 50% credibility interval (CI; boxes) and 95% CI interval (whiskers) of contribution of mangroves, saltmarsh and seagrass to juvenile *F*. *merguiensis* in Deluge Inlet, Cocoa Creek, Doughboy Creek and the two semi-isolated pools in the Ross River floodplain (one surrounded by mangroves and one by saltmarsh).

Stable isotope results show that *F*. *merguiensis* juveniles rely on a range of available sources. The 95% credibility intervals (CI) of mangrove contribution included very low values for all estuaries (low limit of the 95% CI ≤5% in all cases), meaning that it was not possible to positively identify a critical contribution of mangroves. However, mangroves could be important contributors particularly for Doughboy and Cocoa Creeks, where the upper limits of the 95% CI were relatively high (67% and 57% respectively; Fig 5). For Deluge Inlet and for the mangrove-lined pool in the Ross River floodplain, results suggest that mangrove contribution was limited, with the upper bounds of the 95% CI <26% (Fig 5). The stable isotope values used in the mixing models can be found in S3 Table.

Saltmarsh (and its epiphytes) were important for *F*. *merguiensis* juveniles in the saltmarsh-dominated floodplain pool of the Ross River estuary (95% CI: 13–79%; Fig 5), but this importance was limited at Cocoa and Doughboy Creeks (Fig 5), where saltmarsh availability is much lower. Similarly, seagrass had some importance in Deluge Inlet, where extensive seagrass meadows occur, but the importance of this source was likely limited in Cocoa Creek (Fig 5), where seagrass cover is sparse and limited to the creek mouth.

## Discussion

### Limits of the available methods of biomass and production estimates

Despite sampling being confined to a single habitat type, there was consistently high spatial and temporal variability in *F*. *merguiensis* numerical and biomass densities in the Alligator Creek estuary, featuring a consistently large number of samples with zero catches. The reasons for this variability are complex, but not unexpected [20, 53]. Evaluating patterns of abundance of mobile organisms is always difficult because, rather than a few prominent parameters determining how many individuals occur in a particular place at a particular point in time, local abundance is the result of the interaction of a diversity of factors, what has been described as a causal thicket [54]. Causal thickets are characterised by the presence of many interacting and synergistic drivers that change in their relative importance in determining the location and abundance of individuals from time to time and place to place, and that are often strongly aliased and invariably include substantial indeterminacy [55]. Some of the complicating drivers are obvious, e.g. numbers and biomass are simultaneously influenced by emigration, recruitment, within-system movements and mortality, as well as changes in the physical environment such as rainfall-driven salinity depression. The influences of these factors are clear in the present case study. For instance, simultaneous growth and emigration of individuals already in the population, coincident with recruitment of new individuals resulted in changes in biomass density apparently decoupled from changes in numeric density. Other drivers are likely to be important over longer time scales. For instance, this study was conducted during an El Niño event, during which rainfall in North Queensland was well below mean (Bureau of Meteorology, www.bom.gov.au, accessed 09/02/17), so results presented here are unlikely to be representative of those under different climate phases. Indeed, the structure of nekton assemblages in north eastern Australian estuaries [34, 35, 56] and the sources of nutrition that support them [57] are fundamentally different during extended wet versus extended dry periods.

Substantial variations in *F*. *merguiensis* distribution and abundance have been reported by several previous studies. Although juveniles are known to mostly occur along mangrove-lined banks [6, 7], there are considerable variations in density both among and within estuaries [20, 58]. For example, within a system, densities in smaller creeks are often higher than those in the main river [6, 59]. Depth, bank slope and the proximity to mangroves are also important factors affecting densities [7, 59]. Moreover, juveniles can move up to 200 m into mangrove forests at high tide [59, 60] and use the adjacent banks during the low-tide periods when waters are mostly outside of the forests [29, 59, 60], meaning that densities vary through the tidal cycles. Despite their willingness to move into mangroves, at high tides, densities of *F*. *merguiensis* have been found to be higher at creek edges than in the inside of mangrove forests [59, 61].

It is clear that quantifying numerical density, population numbers, biomass density and total biomass of even common mobile estuarine organisms is complex, even if an adequate sampling design is combined with fine-scale mapping. Consequently, although the methodological approach used here was useful to identify important habitat use patterns, the high variability among replicate samples and the logical issues involved mean that for most tropical estuary situations it will be impossible to obtain numerical or biomass density estimates with low variability and for which unambiguous estimates of ‘averages’ can be computed. Additionally, it will usually be equally difficult to determine the particular regulating factors affecting those estimates.

The potential for mobile fauna such as *F*. *merguiensis* to move longitudinally in an estuary over short periods of time creates an important additional problem if the aim is to link increase in biomass to particular areas of the estuary or to particular habitat units. Since biomass density estimates are critical for calculating productivity, and a detailed understanding of movement is necessary to allow that productivity to be attributed to specific habitats, there are likely to be very few cases where biomass increase or productivity can be reliably attributed at the within-estuary scale. In fact, this aligns with current ecological understanding that, rather than being a function of a particular habitat unit, the value of coastal wetlands to fisheries species is a function of an interacting mosaic of habitats that together support life functions [62–64].

It is however important to note that many of the sources of indeterminate variability are related to among-habitat and within-estuary scales. This implies that carefully collected data that cover the whole estuary are likely to provide a means for understanding, addressing and integrating variability, for instance due to movement among reaches. Thus, it is possible that in many cases estimates of whole-of-estuary productivity (i.e. estimates that integrate across the whole estuary), will be more reasonably achievable.

### Evaluating the importance of particular habitat types to prawn nutrition

There was no evidence that mangroves, saltmarsh or other particular wetland habitat types are of critical trophic importance to *F*. *merguiensis* juveniles. Stable isotope results from the current study show that *F*. *merguiensis* juveniles can rely on a range of primary producers as sources of nutrition, and that the importance of the different sources depends on their relative availability, as previously reported for other estuarine species (e.g. [65, 66]). Indeed, the importance of mangrove-derived carbon for *F*. *merguiensis* depends on the extent of mangrove forest in the estuaries, and this importance is significant only in systems where more productive habitats such as seagrass beds are absent [32]. In systems with seagrass beds, even if extensive mangrove forests are present, the importance of mangrove carbon is limited and *F*. *merguiensis* juveniles typically rely mostly on a range of aquatic sources [31, 32, 36].

The lack of evidence that any individual habitat type is critically important to the nutrition of *F*. *merguiensis* juveniles means that the value of the habitats used (e.g. mangroves) is most likely primarily related to physical conditions or structure, rather than to a trophic function. These physical conditions could be a function of the presence of the mangroves themselves, e.g. through the direct use of its physical structure for refuge, and/or related to the provisioning of vast intertidal banks and/or shallow water habitat, factors that have been reported as important predictors of *F*. *merguiensis* catches in North Queensland estuaries [18]. Note that when different habitats are available, *F*. *merguiensis* prefer complex structured mangrove habitats such as mangrove debris and pneumatophores, particularly when in presence of predators [67], a behaviour that has been linked to minimizing predation risks [67, 68]. However, since in our study area mangrove structure is only available for limited time due to the topography and high range (~4 m) of the semi-diurnal tides, it is likely that, when submerged, this structure is used as refuge, but nutrition comes from producers from a range of habitats including mangroves forests, shallow banks and seagrass beds.

The lack of a specific trophic relationship between particular wetland habitat types and *F*. *merguiensis* juveniles adds weight to the argument that productivity outcomes for *F*. *merguiensis* are derived at the whole-of-estuary scale and so it will rarely be possible to make a trophic link between biomass increase and specific wetland units. Consequently, it is likely that productivity is most usefully and validly assessed at the whole-of-estuary scale.

### Value and validity of nekton production estimates at different scales

**Fig 6:**
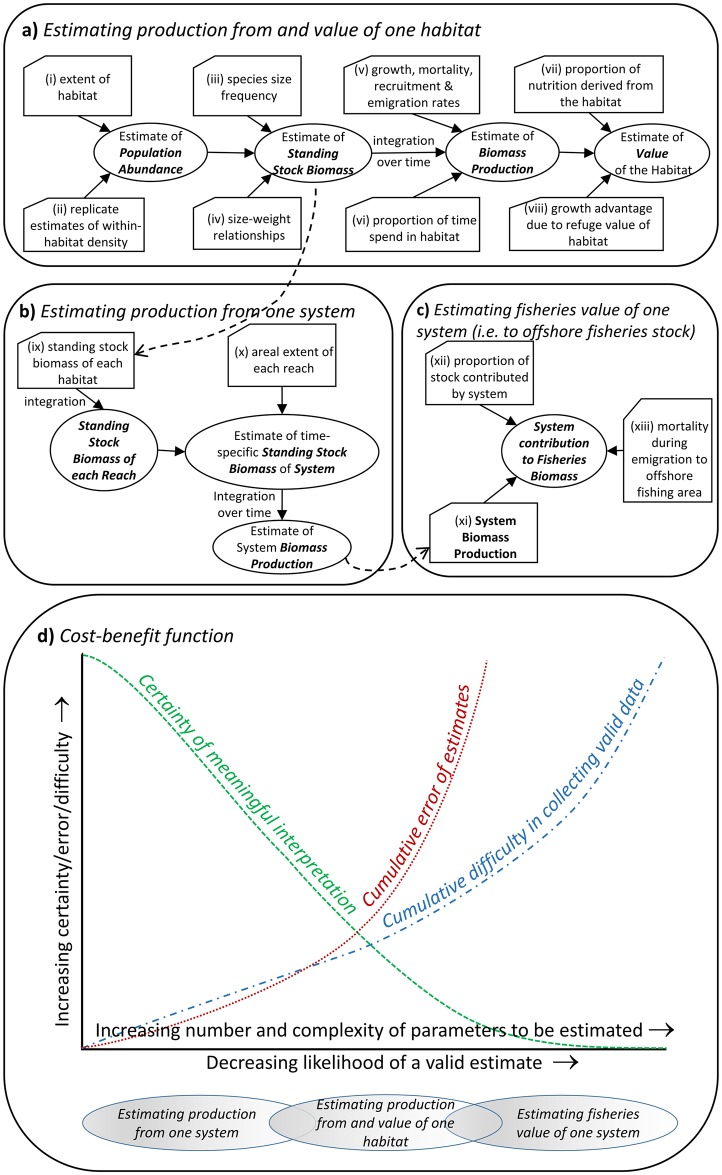
Complexities of estimating production and value. The complexity of estimating (a) production and value from one habitat, (b) production from one system (estuary), (c) fisheries value of one system (measured as contribution to offshore fisheries stock), and (d) indicative cost-benefit function model showing curves for changes in certainty of meaningful interpretation, cumulative error of estimates and cumulative difficulty in collecting valid data, versus the likelihood of a valid estimate. The approximate potential range of estimates (a), (b) and (c) are indicated below the figure.

A diversity of factors need to be taken into account when estimating biomass and production of populations of mobile estuarine nekton such as *F*. *merguiensis*, when evaluating the contribution of particular tidal wetlands to biomass production, and ultimately when linking particular habitat units to the productivity of offshore fisheries stocks (Fig 6). Firstly, detailed sampling of the available habitats at different temporal and spatial scales is critical. However, in many cases this is logistically difficult and time consuming. Indeed, nekton densities [69, 70], biomass and production [27] vary among habitats and depend on a range of factors such as flooding patterns (duration, frequency and depth), the assemblage of habitats, habitat fragmentation (which influences amount of wetland vegetation/water edge), overall arrangement of habitats in the coastal seascape, salinity and temperature [27, 71–75]. Therefore, not only must the area of the different habitats be adequately measured, but the access to these habitats also needs to be considered, and this is influenced by topography, hydrology, and connectivity between the different habitats [76]. Different scales of habitat should also be taken into account (e.g. edge vs. different distances from the edge). All these factors can affect habitat availability and habitat use and value, and should be considered in biomass and production estimation models.

Growth rates and natural mortality rates are other key parameters needed to estimate penaeid production. Growth rates can be estimated using methods such as mark-recapture (e.g. [77, 78]), length-frequency analysis and modal progression (e.g. [10, 21, 79]), and mesocosms (e.g. [80, 81]) and caging (e.g. [69, 82]) experiments. Mortality estimates can also be derived using mark-recapture studies (e.g. [78, 83, 84]) or catch-curve or cohort progression analyses (e.g. [82, 83, 85]). These biological parameters are however difficult to estimate for nekton juveniles in coastal wetlands. For example, cohort analysis is difficult to apply due to long periods of continuous recruitment and when there are multiple waves of recruitment and/or immigration [10, 82]. Also, juveniles of taxa such as penaeids only use estuarine nurseries for relatively short periods of time, and typically use different parts of the estuary as they grow, so different sized juveniles occur in different areas [6, 59, 86] making it difficult to follow cohorts or to relate increase in biomass to the use of particular habitat units. Tag-recapture methods are also difficult to use due to the small size of estuarine juveniles, and because of their high abundances and very high natural mortality rates. Moreover, fecundity [87] and recruitment [17, 86, 88, 89] often vary seasonally and among years; growth rates can vary with temperature [79], salinity and food availability [81]; juvenile densities not only vary seasonally but also through day-night and tidal cycles [90, 91], and mortality rates vary with sizes and among years, habitat type and complexity, and with density [92, 93].

All these and a range of other considerations make estimating production and attributing a value to particular habitats a complex issue. Using our *F*. *merguiensis* example, we could consider three different types or stages of estimates (Fig 6): (i) estimates of production and value of just one type of estuarine habitat (Fig 6a), (ii) estimates of production from one (estuarine) system (Fig 6b), and (iii) estimates of values of one system to the fishery (i.e. to the offshore stock) (Fig 6c). Each of these is dependent on having a range of data and on the quality and validity of those data.

Estimating production from a single habitat requires a diversity of data (Fig 6a), much of it difficult to collect but potentially estimable (e.g. extent of that habitat, species size frequency). Other necessary data (e.g. growth, mortality, recruitment and emigration rates, proportion of time spent in a habitat, etc.) is probably beyond our current abilities and some (e.g. the growth advantage due to refuge or food provision from a habitat) are virtually impossible to collect with any available technology. Moreover, each of the estimates needed to calculate production is associated with a certain level of uncertainty (error), which is often substantial (note for instance the substantial variability in the density estimates seen in Fig 1), each adding to the uncertainty of the combined, final, estimate. This complexity and uncertainty means that both the cumulative difficulty in collecting valid data and the cumulative error of the estimates is high, leading to a low likelihood of a valid estimate and low certainty of meaningful interpretation (Fig 6d).

Estimating the production from one whole system (Fig 6b) is likely to involve a lower level of uncertainty (assuming that prawns remain within the overall estuary for their juvenile phase) because some of the more problematic estimates (e.g. proportion of time spent in each habitat, growth advantage due to refuge value) are not required, and the additional estimates (e.g. areal extent of each estuary reach) will usually be easy to evaluate. Therefore, the certainty of meaningful interpretation and likelihood of a valid estimate are likely to be higher than for estimates of the value of individual habitats (Fig 6d). However, while estimating the production from a whole system is likely to be more feasible than assessing production from individual habitats, such estimates are still sensitively dependent on the quality of data and on the assumptions underpinning the various estimates (e.g. assumptions on distribution, movement, recruitment, migration, size-related mortality, etc.). These requisites need to be exhaustively assessed and the value and reliability of estimates critically appraised before any quantitative use can be made of them.

Indeed, intensive, multidisciplinary, long-term studies would be required to obtain the information that would be needed for accurate productivity estimates. As mentioned above, these would include studies of habitat use, of densities in different habitats and at different scales, and of temporal patterns of movement (including migration and recruitment); estimation of growth rates and natural mortality during residence in different habitats and during different life-history stages; identification of the trophic importance of the habitats accessed throughout ontogeny; and characterization of the effects of the different environmental parameters, such as temperature and salinity, on e.g. growth rates, mortality and movement. These data should then be combined with detailed habitat, topographic, bathymetric and hydrologic mapping, with analyses of tides and inundation patterns, with long-term data on environmental parameters (e.g. rainfall and temperature) and with fisheries catch and effort. It is clear that developing authoritative, robust and reliable estimate value would necessarily involve a very high, long-term, research effort, and could only be (economically) feasible in the case of highly valuable fisheries such as the penaeid prawn fisheries in the Gulf of Mexico, USA (see [27] and [76]). Even then, the caveats discussed above relating to pervasive uncertainty mean that, even at the whole-of-estuary scale, sufficiently reliable estimates may be unachievable.

The final stage of production estimates would be to evaluate the contribution of juveniles from one system to the fisheries stock (for *F*. *merguiensis* this would be in the offshore adult population) (Fig 6c). This is a potentially useful estimate because it allows the attribution of a dollar value to a particular system. However, these estimates require the calculation of some problematic values. For instance, juveniles from several estuaries usually contribute to the same offshore fishery. Moreover, recruitment can vary greatly even among adjacent estuaries due to the hydrological pattern and the spatial arrangement of habitats within systems, and systems within the area [6, 45, 94], meaning that different estuaries will likely contribute differently to the offshore fishery. Within the same system, different cohorts can also contribute differently to the offshore fishery, and this can vary among years [10]. Additionally, the same species can display differences in recruitment patterns to the different fisheries, and can enter the fisheries at different ages and growth phases depending on the system [95]. Some biochemical techniques (e.g. stable isotope analysis) can help determine the proportion of the stock contributed by each system, but this will only be possible where there are measurable differences in biochemical composition among the potential source estuaries. Even more difficult to estimate are mortality rates (and therefore loss of biomass) during migration to the adult grounds and prior to sampling/fishing. The substantial difficulty in estimating all these parameters means that the likelihood of obtaining a valid estimate of the value of one system to the offshore fishery is at least as unlikely as obtaining a valid estimate of production from a single habitat (Fig 6d).

Added to the difficulties associated with valid data collection and parameter estimation, are the critical limits placed on estimation by the presence of pervasive complexity that puts limits on what is possible to predict [55]. For instance, it is now understood that cause and effect are rarely linearly related but rather under the influence of causal thickets [54] and extensively aliased [55]. Additionally, even in well-studied systems there may be pervasive irreducible uncertainty [96]. Thus, it is likely that in many cases detailed levels of estimation will be unachievable.

### Conclusion

Even for a well-studied species such as *F*. *merguiensis* there is still a substantial deficit in the information needed to make precise species-productivity links, with substantial research still needed before the available broad-scale biomass estimates can be converted to valid estimates of total biomass and production. In the example studied, a diversity of potential and actual sources of error and logical problems means that quantification at any scale is at best of uncertain validity and produces estimates that are likely to produce unreliable results if treated as quantitative inputs to production models. In fact, such estimates are probably best treated as indices of relative numerical and biomass densities. Moreover, given the level of uncertainty attached to the estimation of ‘average’ biomass density, and the range of assumptions that need to be made, it is unclear if such estimates are of substantially greater value than simple measures such as Probability of Encounter [58]. It is even more difficult to take the next step: relating the estimates to particular habitats. Being able to estimate productivity and attribute it sensitively and validly to particular units (a wetland or an estuary) is crucial if estimates of fisheries (monetary) value are to be used to inform management decisions and support ecosystem restoration efforts targeted at particular habitat units. However, for many situations it is likely to be unattainable with current technologies, and those likely to come on line in the short to medium term.

Results from the present study have substantial implications for conservation, restoration and management of tropical coastal wetlands, and of the fisheries that rely on them. The manifest difficulties in determining and assigning value to individual habitats, the vast amount of information needed to support viable estimates, and the uncertainty of current paradigms [97], implies that extreme caution is required if estimates of the value of fisheries production are to be used as a basis for decision making. In fact, quantification of fisheries production does not represent a simple solution to valuing coastal wetlands because the complexity of issues involved mean that, in many or even most cases, it will not be possible to produce unambiguous and valid estimates. Although many factors complicate assigning value to habitats challenging, the most fundamental is the mobility of a large proportion of marine fisheries species relative to the time over which biomass is accumulated. Consequently, there is an underlying life-style-associated gradient that sets a base level on what is possible, with very site-attached taxa (e.g. bivalves/gastropods) providing the greatest opportunity for successful attribution of value to specific habitat units, and the most mobile (e.g. many shrimps and fish) integrating their acquisition of biomass across many habitats, almost invariably rendering habitat-specific attribution untenable. Over and above this base level constraint, the range of additional factors that need to be considered means that, from a practical perspective, the ability to confidently and validly attribute a monetary value will be limited in most cases. Consequently, it is clear that a substantial effort to increase the extent, detail and validity of coastal fisheries science is needed before habitat valuation based on fisheries production can provide an objective basis for the prioritisation of management, conservation and restoration actions that can be relied on to confidently produce measurable beneficial outcomes.

## Supporting information

S1 Table *Fenneropenaeus merguiensis* captures. Data on *Fenneropenaeus merguiensis* captures (number and weight of prawns per net) for the downstream (DS), mid-estuary (MS) and upstream (US) reaches of Alligator Creek during each of the eleven trips.
(XLSX)

S2 Table *Fenneropenaeus merguiensis* size structure. *Fenneropenaeus merguiensis* size structure for each trip (T1-T11) for the downstream (DS), mid-estuary (MS) and upstream (US) reaches of Alligator Creek. Data was binned into 5 mm size classes.
(XLSX)

S3 Table Stable isotope data used in the mixing models. Carbon (δ^13^C) and nitrogen (δ^15^N).
(XLSX)
