# Correction: Estimating the value of tropical coastal wetland habitats to fisheries: Caveats and assumptions

**DOI:** 10.1371/journal.pone.0223797

**Published:** 2019-10-07

**Authors:** Kátya G. Abrantes, Marcus Sheaves, Jakob Fries

There are errors in the Funding statement. The correct Funding statement is as follows: Researcher Marcus Sheaves was supported by the Marine Biodiversity Hub through funding from the Australian Government's National Environmental Science Program. The funders had no role in study design, data collection and analysis, decision to publish, or preparation of the manuscript.
